# Associations between meteorological factors and scrub typhus incidence: a systematic review and meta-analysis of linear and nonlinear dose–response relationships

**DOI:** 10.1186/s41182-025-00819-0

**Published:** 2025-10-29

**Authors:** Shu Yang, Shu Yang, Jun Guo, Peng Li, Yuling Xu, Fei Hu, Yiting Cui, Ai Peng, Yangqing Liu, Yibing Fan, Shihui Peng, Hui Li, Peng Huang

**Affiliations:** 1 Center for Evidence-Based Medicine, Jiangxi Provincial Key Laboratory of Disease Prevention and Public Health, School of Public Health, Jiangxi Medical College, Nanchang University, Nanchang, 330019 China; 2 The Collaboration Unit for Field Epidemiology of State Key Laboratory of Infectious Disease Prevention and Control, Nanchang Center for Disease Control and Prevention, Nanchang, 330038 China; 3 Longnan Center for Disease Control and Prevention, Ganzhou, 341700 Jiangxi China; 4 Nanfeng Center for Disease Control and Prevention, Fuzhou, 344500 Jiangxi China; 5 Department of Cardiac Surgery, The Second Affiliated Hospital, Jiangxi Medical College, Nanchang University, Nanchang, 330000 China; 6 Department of Cancer Medical Center, The First Affiliated Hospital, Jiangxi Medical College, Nanchang University, Nanchang, 330019 China

**Keywords:** Scrub typhus, Meteorological factors, Weather, Risk factor, Meta-analysis

## Abstract

**Background:**

Scrub typhus is an important zoonotic disease with rising incidence globally. Meteorological factors may influence its transmission dynamics. However, inconsistencies across studies and limited quantitative evidence highlight the need for further investigation.

**Objective:**

To systematically evaluate the linear and nonlinear associations between various meteorological factors and scrub typhus incidence, explore lagged effects and potential sources of heterogeneity, and analyze inconsistencies in existing findings.

**Method:**

We searched PubMed, Scopus, Web of Science, and Embase for studies published up to February 2025. Relevant articles were identified based on predefined inclusion and exclusion criteria. We conducted linear meta-analyses to assess the effects of unit changes in meteorological factors and dose–response meta-analyses to evaluate cumulative lagged risks across different exposure levels. Subgroup and sensitivity analyses were conducted to explore sources of heterogeneity and assess result robustness.

**Results:**

Seventeen studies were included, covering China, Korea, Laos, and India. Eleven studies contributed to linear meta-analyses, and six to dose–response analyses. Ambient temperature (RR 1.08, 95% CI 1.02–1.16), land surface temperature (RR 1.06, 95% CI 1.02–1.09), precipitation (RR 1.01, 95% CI 1.01–1.02), relative humidity (RR 1.07, 95% CI 1.04–1.11), and atmospheric pressure (RR 1.06, 95% CI 0.91–1.23) were positively associated with the risk of scrub typhus. Wind speed (RR 0.59, 95% CI 0.49–0.71) and sunshine duration (RR 0.92, 95% CI 0.77–1.10) exhibited negative associations. Dose–response meta-analysis revealed inverted U-shaped relationships for ambient temperature and relative humidity, and a unimodal pattern for precipitation, with risk increasing continuously at high levels. Significant lag effects were observed: precipitation had the most immediate effect (lag0: RR 1.05), while ambient temperature (lag1: RR 1.18) and relative humidity (lag2: RR 1.28) peaked with a 1- to 2-month delay. Geographic variation was identified as a major source of between-study heterogeneity.

**Conclusion:**

Although this study has certain limitations, including the small number of included studies, their concentration mainly in China, and the presence of substantial heterogeneity, the results provide evidence of linear and nonlinear associations between meteorological factors and scrub typhus incidence, and highlight the roles of geographical variation and lag effects. These findings offer quantitative evidence and scientific support for disease prevention and control in the context of climate change.

**Supplementary Information:**

The online version contains supplementary material available at 10.1186/s41182-025-00819-0.

## Introduction

Scrub typhus is an acute febrile naturally occurring epidemic disease caused by infection with *Orientia tsutsugamushi*, and is primarily transmitted to humans through the bite of infected chigger larvae, mainly of the *Leptotrombidium* genus [1]. This disease is widely prevalent globally, with over one billion people estimated to be at potential risk and more than one million cases reported annually [2]. Infected individuals often present with a range of clinical symptoms [3], including high fever, headache, lymphadenopathy, and eschar formation [4]. Without timely diagnosis and appropriate treatment, scrub typhus may rapidly progress to systemic complications involving multiple organs and can lead to fatal outcomes [5].

The “tsutsugamushi triangle” in the Asia–Pacific region has long been regarded as the traditional endemic area for scrub typhus, spanning from Japan to Afghanistan and extending southward to northern Australia and northward to the Russian Far East coast [6]. It remains the most severely affected region worldwide. Although historically confined to this geographic range, emerging foci have been increasingly reported in recent years, including in Chile and Peru in South America, parts of the Middle East, and several African countries [7, 8]. These findings indicate that the geographic distribution of scrub typhus is expanding beyond its conventional boundaries, suggesting a growing risk of global spread. As the geographic boundaries of scrub typhus continue to expand, its prevalence and disease burden in human populations are also becoming clearer. Research [9] indicates widespread latent or past infections exist within healthy populations, with a combined seropositivity rate of 10.7%, rising to 22.6% among febrile patients. Marked differences in incidence have also been reported across countries: China has documented the largest number of cases, while South Korea and Thailand exhibit relatively high incidence rates. Collectively, these findings indicate that scrub typhus has emerged as a significant global public health threat.

The transmission of scrub typhus involves complex interactions among vector–host ecology, environmental factors, and human social activities. As a quintessential vector-borne disease, scrub typhus is transmitted to humans through bites of infected chigger mite larvae. The chigger mite life cycle comprises four stages: egg, larva, nymph, and adult [10]. Only the larval stage is parasitic**,** transmitting pathogens through skin penetration during feeding [11]. The primary reservoir hosts are small mammals, predominantly rodents [10].

Meanwhile, the transmission dynamics of scrub typhus are closely associated with human activities. Studies [9] have shown that the main risk factors include agricultural work, exposure to specific vegetation, frequent outdoor activities [12, 13], and contact with rodents or domestic animals, all of which increase the likelihood of exposure to chigger mites. In addition, people living near grasslands, vegetable gardens, or ditches are at higher risk of infection, as these environments provide ideal habitats for mite propagation [14–17].

Additionally, increasing attention has been paid to the regulatory role of meteorological factors in infectious diseases. Numerous studies have explored the associations between meteorological variables and the incidence of scrub typhus, and some systematic reviews have summarized the existing evidence [18]; however, there is still a lack of quantitative meta-analyses. Furthermore, inconsistencies remain among published studies. For instance, while most studies have reported ambient temperature and scrub typhus incidence [19–25], a study conducted in India [26] reported an inverse relationship. Similarly, although studies from China suggest that higher land surface temperature increases scrub typhus risk [23, 27, 28], a study from Korea [29] found a negative association. With respect to precipitation, most studies have identified a positive correlation [19–24, 26, 27, 30, 31], though variations exist. For example, a study in China [32] found opposite associations between precipitation, relative humidity, and scrub typhus risk in northern and southern regions. Even within the same country, associations between relative humidity, wind speed, and scrub typhus incidence have been inconsistent across different studies [19, 33–35]. Furthermore, evidence concerning sunshine duration and atmospheric pressure remains limited and contradictory [25, 31, 36–39].

These inconsistencies across studies may compromise the accuracy and generalizability of predictive models developed from single studies or specific locations, thereby reducing the effectiveness of scrub typhus early warning systems based on meteorological factors. In addition, such variability poses challenges for governments in formulating unified prevention and control measures, making it difficult to apply strategies developed in one region to others. Addressing these inconsistencies is therefore essential not only for improving the stability and reliability of predictive models, but also for providing a stronger evidence base for the development of more scientific and widely applicable public health interventions.

To address these inconsistencies, we conducted a systematic review and meta-analysis to quantitatively synthesize the available evidence. We performed a linear meta-analysis to estimate the effects of unit changes in meteorological factors on scrub typhus incidence, and for the first time, we conducted a dose–response meta-analysis to explore the cumulative lag effects of meteorological factors. Subgroup analyses were also performed to examine the influence of country, lag structure, and model type. By integrating the most recent evidence, our study aims to explain the inconsistencies among previous studies and mitigate the bias inherent in individual studies. This work facilitates the systematic evaluation of meteorological influences on scrub typhus incidence, explores underlying mechanisms, provides direction for future research, and offers a scientific basis for scrub typhus prevention and control.

## Material and methods

This study was conducted as a systematic review and meta-analysis to quantitatively assess the association between meteorological factors and the incidence of scrub typhus. The reporting of the study followed the Preferred Reporting Items for Systematic Reviews and Meta-Analyses (PRISMA) guidelines. This systematic review and meta-analysis has been registered in PROSPERO (CRD420251068298).

### Search strategy

A comprehensive literature search was performed using PubMed, Scopus, Web of Science, and Embase to identify eligible studies published between February 5, 2015 and February 5, 2025. In addition, the reference lists of all included studies and relevant review articles were manually screened to identify any additional potentially relevant publications.

**Fig. 1:**
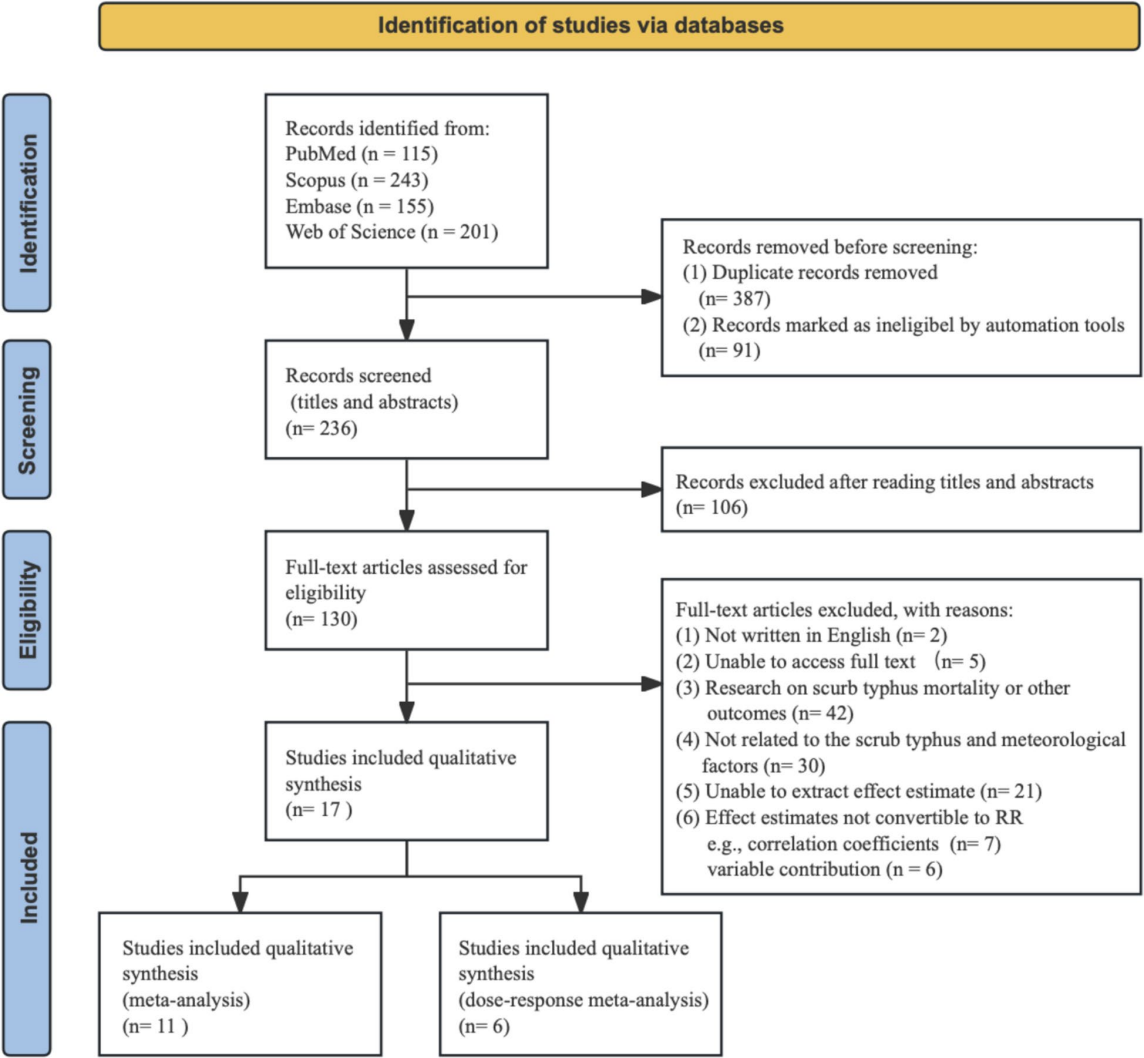
PRISMA flow diagram of study selection

The search strategies were based on the combinations of keywords related to meteorological factors (“temperature” OR “humidity” OR “rain” OR “rainfall” OR “precipitation” OR “wind speed” OR “wind velocity” OR “sunshine” OR “sunlight” OR “sunshine duration” OR “atmospheric pressure” OR “air pressure” OR “barometric pressure” OR “climate” OR “weather” OR “meteorolog*”) and scrub typhus (“scrub typhus” OR “*Orientia tsutsugamushi*” OR “tsutsugamushi disease” OR “tsutsugamushi Fever”), with the two groups of terms combined using the Boolean operator “AND”. We supplemented the computerized search with a manual search of the bibliographies of all retrieved publications to identify other potentially eligible studies. The search was restricted to studies on humans, published in English. Grey literature was not included because of difficulties in extracting standardized effect estimates and concerns about data completeness and quality. The study screening process is shown in Fig. 1, and the full search strategies for each database are provided in Supplementary Table S1.

Two independent researchers (Yu and Hu) performed the literature search separately, with a third one for validation (Yang). Discrepancies were resolved through discussion and consensus.

### Study inclusion and exclusion criteria

In the initial screening phase, titles and abstracts were reviewed to identify potentially relevant studies. Studies were included if they met the following criteria:

1. The study population was human, and the outcome of interest was the incidence of scrub typhus;
2. The study investigated the exposure of interest between one or more meteorological factors (e.g., temperature, humidity, precipitation) and scrub typhus incidence;
3. The study included a comparator, defined as variations in meteorological exposures across different levels or ranges (e.g., per 1 °C increase, per 1 mm of precipitation, or multiple exposure levels with sufficient data to allow comparison);
4. The study reported relative risk (RR), incidence rate ratio (IRR), hazard ratio (HR), or other effect estimates convertible to RR and corresponding 95% confidence intervals, based on unit changes in meteorological variables; or reported multiple exposure levels with sufficient data to calculate logRR and its standard error (SE);
5. The study was original research with full-text availability and a design of either a cohort study or a case–control study.

Studies were excluded if they met any of the following criteria:

1. The article was not published in English;
2. The article was a duplicate publication;
3. The study did not report extractable or convertible effect size data.

### Data extraction

We extracted the following information from each study that met the inclusion criteria: first author, publication year, study region, data sources, meteorological exposure variables, effect estimates with 95% confidence intervals (CI), and statistical models used.

For studies reporting multiple exposure levels (e.g., different temperature, humidity, or precipitation values) and their corresponding effect estimates (e.g., RR and 95% CI), we extracted all dose-specific data points. If the exposure–response relationship was presented graphically (e.g., curves derived from distributed lag nonlinear models [DLNM]), we used the *WebPlotDigitizer* web application (v4.8; Automeris, https://automeris.io/WebPlotDigitizer/) to extract the RR and CI for each dose point and converted them into logRR and standard error (SE) for use in the dose–response meta-analysis.

Given the variation in lag structures used across studies (e.g., single-day vs. cumulative lag), we applied the following rules to standardize effect estimates for pooling: (1) if only one lag period was reported, we used that estimate; (2) if multiple lag periods were reported, we prioritized: the lag specified as primary by the authors, the lag with statistically significant results, or the lag with the largest effect estimate.

For studies reporting nonlinear or threshold effects, we treated each exposure level as a separate data point. If multiple model estimates were presented, we prioritized the final model specified by the authors; if unspecified, we selected the model with the most covariate adjustments.

In studies involving multiple geographic regions or subpopulations, we extracted the overall estimate when available. If only subgroup estimates were provided, we treated each subgroup as an independent entry to prevent any single study from exerting excessive weight in the meta-analysis.

### Quality assessment of studies in the meta-analysis

The Newcastle Ottawa Scale (NOS) was applied to assess the quality of case–control and cohort studies, as well as the quality of the selected articles. The NOS included eight items in three dimensions, including the selection, comparability, and the outcome. Two investigators (Xu and Hu) independently assessed the quality of the items following the NOS guidelines, while a third arbitrator (Yang) resolved any disagreements between the two investigators. Articles with a NOS score of 6 or higher were classified as moderate or high quality.

### Statistical methods

All statistical analyses were conducted using R software (version 4.4.0). Two types of meta-analyses were performed in this study: a linear main-effect meta-analysis and a dose–response meta-analysis, aiming to evaluate the linear and potential nonlinear associations between meteorological factors and the risk of scrub typhus.

#### Linear meta-analysis

To evaluate the linear association between meteorological factors and the risk of scrub typhus, we extracted effect estimates reported per unit increase in exposure (e.g., per 1 °C rise in temperature, per 1 mm increase in precipitation, or per 1% increase in relative humidity), including relative risks (RRs), incidence rate ratios (IRRs), or hazard ratios (HRs), along with their 95% confidence intervals (CIs). Given that scrub typhus is a low-incidence disease, these measures were treated equivalently as RRs in the meta-analysis. All effect estimates were transformed into natural logarithms (logRRs) and corresponding standard errors (SEs) prior to pooling.

Between-study heterogeneity was assessed using Cochran’s Q test and the *I*^2^ statistic. A fixed-effects model was applied when heterogeneity was low (*I*^*2*^ < 50%), whereas a random-effects model using the DerSimonian–Laird method was employed when substantial heterogeneity was present (*I*^*2*^ ≥ 50%) to estimate the overall association between meteorological exposures and scrub typhus risk with 95% CIs, while incorporating potential variability across studies. The DerSimonian–Laird method [40] is widely recommended in epidemiological meta-analyses, as it provides a robust estimation of pooled effects while accounting for between-study variability.

#### Dose–response meta-analysis

For studies reporting multiple exposure levels (e.g., different values of temperature, humidity, or precipitation) and their corresponding effect sizes, a dose–response meta-analysis was conducted to explore linear or nonlinear exposure–response relationships. For studies presenting results graphically (e.g., DLNM curves), the *WebPlotDigitizer* web application was used to extract RR values and their 95%CIs at each exposure level.

The dose–response analysis was performed using the two-stage random-effects model proposed by Greenland and Longnecker [41]. In the first stage, trend estimates and covariance matrices were reconstructed based on the reported data; in the second stage, a multivariate random-effects meta-analysis was conducted to pool the trend estimates, allowing for between-study heterogeneity.

The exposure–response relationship was modeled using restricted cubic splines, with knots placed at the 10th, 50th, and 90th percentiles of the exposure distribution. This knot placement strategy is commonly recommended in dose–response meta-analyses, as it provides sufficient model flexibility while minimizing the risk of overfitting [42]. A likelihood ratio test was used to compare the linear model with the spline model to assess the presence of nonlinearity. Between-study heterogeneity was assessed using Cochran’s *Q* test, the *I*^*2*^ statistic, and the tau-squared (*τ*^*2*^) estimate.

#### Subgroup and sensitivity analysis

To explore potential sources of heterogeneity, subgroup analyses were conducted based on potential modifiers such as region, lag structure, and model type. Considering that multiple included studies focused on lagged effects and reported different lag times, we incorporated the reported point lag effect estimates into subgroup analyses, where “lag 0” represented the immediate effect, and “lag 1” and “lag 2” corresponded to delays of one month and two months, respectively. Between-group differences were assessed using the *Q* test, with *p*-values < 0.1 considered statistically significant.

Sensitivity analyses were performed using the leave-one-out method, sequentially excluding each individual study to evaluate its impact on the overall effect estimate and heterogeneity. If the exclusion of any single study did not materially alter the pooled estimates or heterogeneity, the results were considered robust.

#### Publication bias

To assess publication bias, funnel plots were first visually inspected. Egger’s weighted regression test and Begg’s rank correlation test were subsequently performed for statistical evaluation. For analyses with fewer than nine studies, funnel plots were not generated. All statistical tests were two-sided, with *p*-values < 0.05 considered statistically significant.

## Results

### Study selection and characteristics

In the initial search, a total of 714 articles were retrieved. After removing duplicates, 236 unique records remained for screening. During the title and abstract screening stage, 106 articles were excluded due to irrelevant outcomes (not related to scrub typhus incidence), exposures unrelated to meteorological factors, or study designs that did not meet inclusion criteria.

Subsequently, 130 full-text articles were assessed for eligibility. Furthermore, 113 were excluded based on predefined criteria, including lack of extractable or convertible effect estimates (e.g., relative risks), unavailability of full text, or outcomes unrelated to incidence. Although many studies examined the association between meteorological factors and scrub typhus, 13 lacked usable effect estimates—7 provided only correlation coefficients and 6 presented variable importance scores from predictive models, which are not suitable for meta-analysis.

Finally, 17 studies were included in the qualitative synthesis. Among them, 11 studies were eligible for the linear main meta-analysis [19–23, 26, 27, 29, 32–34], and 6 studies were included in the dose–response meta-analysis [24, 31, 43–46]. The full selection process is shown in Fig. 1.

The included studies were published between 2016 and 2025 and were conducted in China, Korea, Laos, and India. The meteorological exposures investigated primarily included temperature, precipitation, relative humidity, wind speed, atmospheric pressure, and sunshine duration. All studies used scrub typhus incidence as the primary outcome. Case data were mainly obtained from national Centers for Disease Control and Prevention (CDCs) or the National Notifiable Infectious Disease Reporting Information System (NNIDRIS), while meteorological data were primarily sourced from local meteorological bureaus or national meteorological data platforms. Detailed study characteristics are summarized in Table 1.

**Table 1:** Characteristics of the included studies on the associations between meteorological factors and the risk of scrub typhus

Author (year)	Location	Study period	Sources of case data	Climate data source	Meteorological factors	Statistical method		Inclusion in meta-analysis	Quality
						Effect size	Statistical model		
Wu et al. 2016 [19]	China	2006–2014	China CDC	Chinese Academy of Meteorological Sciences	Temperature Relative humidity Precipitation	IRR	Spatial regression	Linear meta-analysis	Moderate
Sun et al. 2017 [20]	China	2006–2014	China CDC	China Meteorological Data Sharing Service System	Temperature Precipitation Relative humidity Atmospheric pressure Wind velocity	IRR	Multilevel negative binomial regression	Linear meta-analysis	High
Wei et al. 2017 [27]	China	2006–2014	Chinese Communicable Disease Network	Chinese Bureau of Meteorology	Land surface temperature Relative humidity Precipitation	RR	Distributed lag nonlinear model (DLNM)	Linear meta-analysis	Moderate
Kang & Choi 2018 [33]	Korea	2010–2014	Korea CDC	Korea Meteorological Administration	Temperature Relative humidity Precipitation	RR	Bayesian zero-inflated Poisson spatiotemporal model	Linear meta-analysis	High
Kim & Kim 2018 [29]	Korea	2009–2013	Korea CDC	Korea Meteorological Administration	Land surface temperature Precipitation	RR	Bayesian hierarchical spatial model (BYM)	Linear meta-analysis	High
Yao et al. 2019 [21]	China	1980–2013	China CDC	China Meteorological Data Sharing Service System	Temperature Precipitation Relative humidity Sunshine duration	HR	Cox proportional hazards model	Linear meta-analysis	High
Lu et al. 2021 [46]	China	2006–2018	Chinese National Notifiable Disease Report System	Guangzhou Meteorological Bureau	Temperature Precipitation Relative humidity Wind velocity Atmospheric pressure Sunshine duration	RR	DLNM	Dose–response meta-analysis	Moderate
Roberts et al. 2021 [22]	Laos	2003–2017	Mahosot Hospital	TuTiempo	Temperature Precipitation Relative Humidity Wind speed	Regression coefficient	Multiple linear regression	Linear meta-analysis	High
Luo et al. 2022 [45]	China	2006–2018	Chinese National Notifiable Disease Report System	National Meteorological Data Sharing Center	Temperature Precipitation Relative Humidity Sunshine duration	RR	DLNM	Dose–response meta-analysis	High
Han et al. 2023 [24]	China	2006–2020	National Notifiable Infectious Disease Reporting Information System	China Meteorological Data Sharing Service System	Temperature Precipitation Relative Humidity Wind velocity Atmospheric pressure Sunshine duration	RR	DLNM	Dose–response meta-analysis	High
Li et al. 2023 [23]	China	2012–2020	Chinese National Notifiable Infectious Disease Reporting Information System	China Meteorological Data Service Centre	Temperature Precipitation Relative Humidity Wind velocity Atmospheric pressure Sunshine duration	**IRR**	Univariate negative binomial regression	Linear meta-analysis	High
Wei et al. 2023 [34]	China	2010–2020	China CDC	China Meteorological Data Service Center	Temperature Land surface temperature Precipitation Relative Humidity Wind speed Atmospheric pressure Sunshine duration	**IRR**	Bayesian spatial model and boosted regression trees (BRT)	Linear meta-analysis	High
Chang et al. 2024 [31]	Korea	2001–2019	Korea CDC	Korea Meteorological Administration	Temperature Precipitation	**RR**	DLNM	Dose–response meta-analysis	High
D'Cruz et al. 2024 [26]	India	2005–2020	Christian Medical College	Regional Meteorological Centre	Temperature Precipitation Relative Humidity	Regression coefficient	DLNM	Linear meta-analysis	Moderate
Luo et al. 2024 [44]	China	2010–2019	Chinese National Notifiable Infectious Disease Reporting Information System	China Meteorological Data Service Center	Temperature Precipitation Relative Humidity	**RR**	DLNM	Dose–response meta-analysis	Moderate
Pan et al. 2024 [43]	China	2008.1–2021.12	National Disease Surveillance Information Management System	National Meteorological Science Data Center	Temperature Precipitation Relative Humidity Wind speed Atmospheric pressure Sunshine duration	**RR**	DLNM	Dose–response meta-analysis	High
Qian et al. 2024 [32]	China	2006.1–2018.12	China CDC	China Meteorological Data Sharing Service System	Temperature Precipitation Relative Humidity Wind speed Sunshine duration	**RR**	Bayesian space–time hierarchical model(BSTHM)	Linear meta-analysis	High

As shown in Table S2, all included studies in this review scored 6 or above out of a maximum of 9 points, indicating an overall moderate to high methodological quality.

### Linear meta-analysis

#### The overall effect

**Fig. 2:**
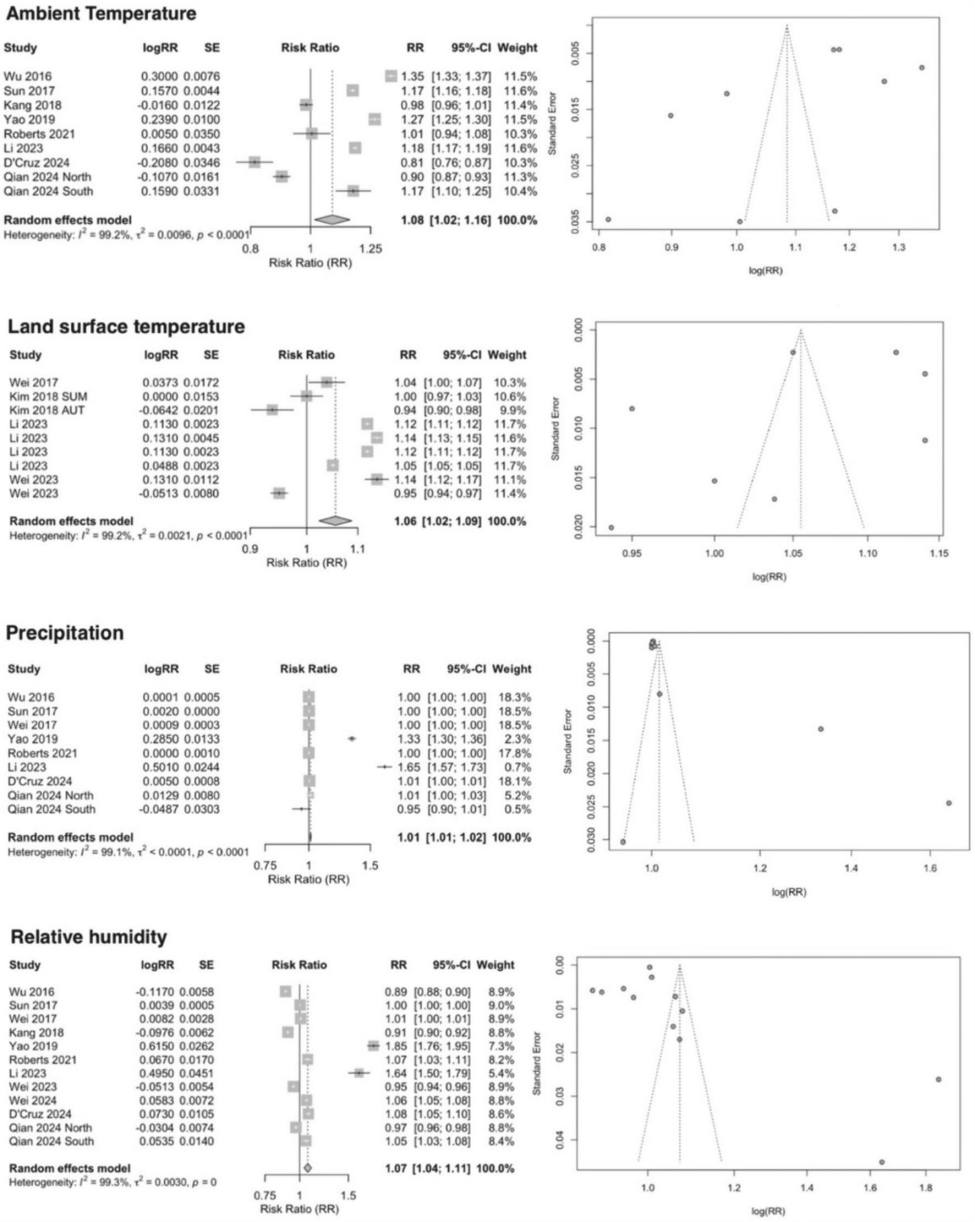
Forest plot and funnel plot showing the ambient temperature (*n* = 9); land surface temperature (*n* = 9); precipitation (*n* = 9); relative humidity (*n* = 12) on the risk of scrub typhus

**Fig. 3:**
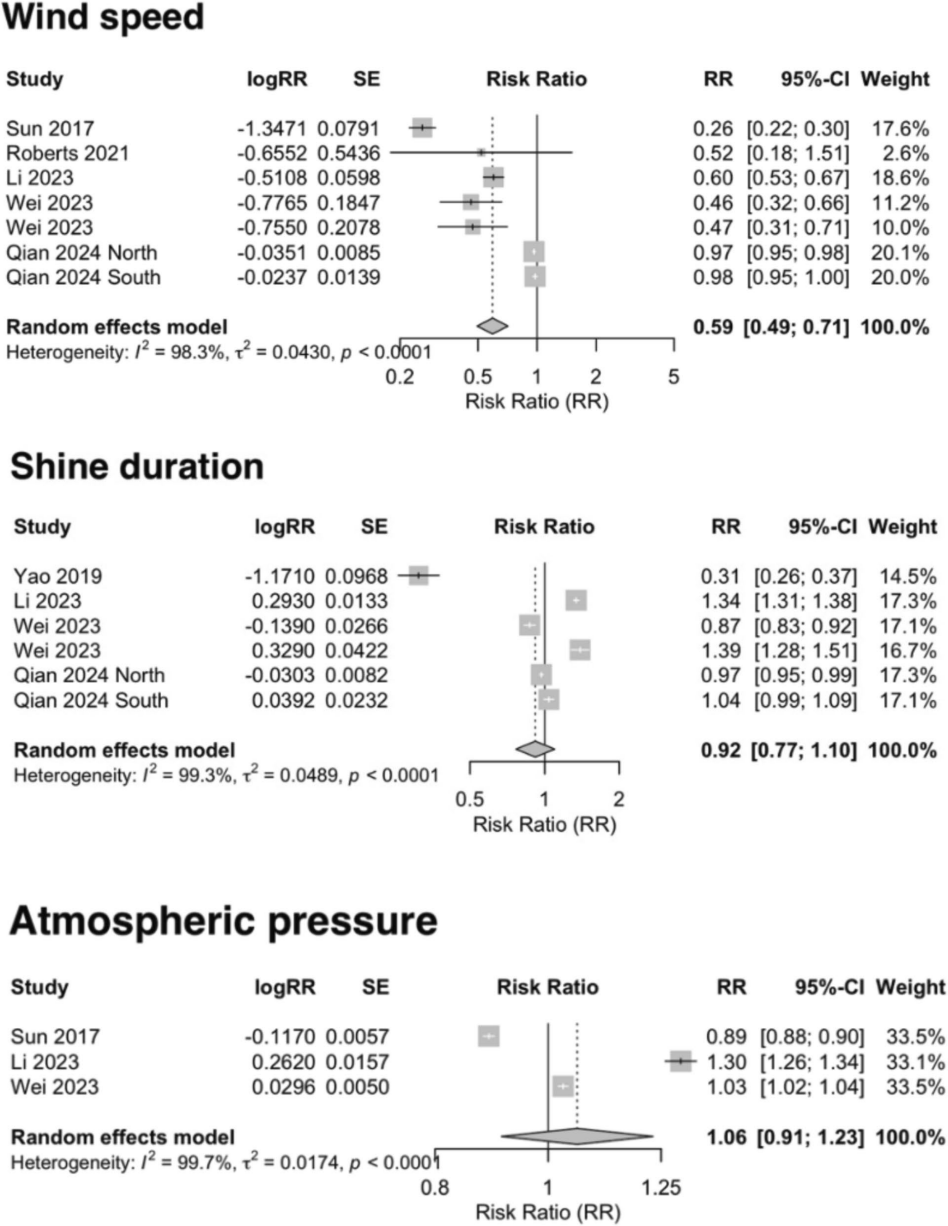
Forest plot showing the wind speed (*n* = 7); sunshine duration (*n* = 6); atmospheric pressure (*n* = 3) on the risk of scrub typhus

During the linear dose–response meta-analysis, we observed substantial heterogeneity among the included studies (ambient temperature: *I*^*2*^ = 99.2%; land surface temperature: *I*^*2*^ = 99.2%; precipitation: *I*^*2*^ = 99.1%; relative humidity: *I*^*2*^ = 99.3%; wind speed: *I*^*2*^ = 98.3%; sunshine duration: *I*^*2*^ = 99.3%; atmospheric pressure: *I*^*2*^ = 99.7%). Therefore, a random-effects model was employed to obtain more robust effect estimates. The high heterogeneity across studies may reduce the reliability of the pooled effect estimates. To ensure robustness, we interpreted the pooled results with caution and evaluated them in conjunction with findings from subgroup and sensitivity analyses. Figures 2 and 3 present the forest plots summarizing the effect estimates for all meteorological factors and the funnel plots for those with a sufficient number of studies to assess potential publication bias.

The meta-analysis results indicated that ambient temperature (RR 1.08, 95% CI 1.02–1.16), land surface temperature (RR 1.06, 95% CI 1.02–1.09), precipitation (RR 1.01, 95% CI 1.01–1.02), relative humidity (RR 1.07, 95% CI 1.04–1.11), and atmospheric pressure (RR 1.06, 95% CI 0.91–1.23) were positively associated with the risk of scrub typhus, while wind speed (RR 0.59, 95% CI 0.49–0.71) and sunshine duration (RR 0.92, 95% CI 0.77–1.10) showed negative associations. It is noteworthy that the pooled effects of atmospheric pressure and sunshine duration on scrub typhus risk did not reach statistical significance, suggesting that current evidence is insufficient to support a definitive association.

#### Subgroup analysis

To further explore the sources of heterogeneity and examine how different study characteristics may influence the overall effect estimates, we conducted subgroup analyses for average temperature, land surface temperature, precipitation, relative humidity, and wind speed based on region, lag type, and model type (see Tables 2, 3, 4, 5, 6 and 7). Due to the limited number of studies available on atmospheric pressure, subgroup analyses were not performed for this variable.

**Table 2:** Subgroup analysis for the association between ambient temperature and scrub typhus

Subgroup types	n	Heterogeneity test		Pooled estimate	
		*I*^*2*^	*P*	RR (95%CI)	*P*
**Regions**					
China	6	99.2%	< 0.0001	1.17 (1.09–1.25)	< 0.0001
Korea	1	NA		0.98 (0.96–1.01)	0.214
Laos	1	NA		1.01 (0.94–1.08)	0.743
India	1	NA		0.81 (0.76–0.87)	< 0.001
**Lag group**					
Lag 0	7	99.4%	< 0.0001	1.06 (0.92–1.21)	0.411
Lag 1	2	53.4%	0.143	1.18 (1.16–1.19)	< 0.001
**Model type**					
Multivariable analysis	6	99.3%	< 0.0001	1.09 (0.99–1.20)	0.073
Univariable analysis	3	99.2%	< 0.0001	1.07 (0.89–1.30)	0.472
**Overall**	9	99.2%	< 0.0001	1.08 (1.02–1.16)	0.015

**Table 3:** Subgroup analysis for the association between land surface temperature and scrub typhus

Subgroup types	n	Heterogeneity test		Pooled estimate	
		*I*^*2*^	*P*	RR (95%CI)	*P*
**Regions**					
China	7	99.4%	*p* < 0.0001	1.08 (1.04–1.12)	< 0.0001
Korea	2	84.5%	0.0110	0.97 (0.91–1.03)	0.3371
**Lag group**					
Lag 0	4	97.9%	< 0.0001	1.02 (0.94–1.11)	0.5998
Lag 1	2	0%	1.000	1.14 (1.13–1.15)	< 0.0001
Lag 2	1	NA		1.12 (1.11–1.12)	< 0.0001
Lag 3	2	99.3%	< 0.0001	1.00 (0.91–1.10)	0.9848
**Model type**					
Multivariable analysis	4	98.4%	< 0.0001	1.00 (0.91–1.21)	0.9309
Univariable analysis	5	99.3%	< 0.0001	1.09 (1.06–1.13)	< 0.0001
**Overall**	9	99.2%	< 0.0001	1.06 (1.02–1.09)	0.0007

**Table 4:** Subgroup analysis for the association between precipitation and scrub typhus

Subgroup types	n	Heterogeneity test		Pooled estimate	
		*I*^*2*^	*P*	RR (95%CI)	*P*
**Regions**					
China	7	99.3%	< 0.0001	1.02 (1.01–1.03)	< 0.0001
Laos	1	NA		1.00 (1.00–1.00)	NA
India	1	NA		1.01 (1.00–1.01)	NA
**Lag group**					
Lag 0	5	99.1%	< 0.0001	1.05 (1.03–1.07)	< 0.0001
Lag 2	3	99.5%	< 0.0001	1.04 (1.03–1.06)	< 0.0001
Lag 4	1	NA		1.00 (1.00–1.00)	NA
**Model type**					
Multivariable analysis	3	93.4%	< 0.0001	1.00 (0.00–1.00)	0.3056
Univariable analysis	6	99.4%	< 0.0001	1.03 (1.03–1.04)	0.1396
**Overall**	9	99.1%	< 0.0001	1.01 (1.01–1.02)	< 0.0001

**Table 5:** Subgroup analysis for the association between relative humidity and scrub typhus

Subgroup types	n	Heterogeneity test		Pooled estimate	
		*I*^*2*^	*P*	RR (95%CI)	*P*
**Regions**					
China	9	99.4%	< 0.0001	1.09 (1.05–1.13)	< 0.0001
Korea	1	NA		0.91 (0.90–0.92)	< 0.0001
Laos	1	NA		1.07 (1.03–1.11)	< 0.0001
India	1	NA		1.08 (1.05–1.10)	< 0.0001
**Lag group**					
Lag 0	8	99.4%	< 0.0001	1.06 (0.99–1.14)	0.1005
Lag 2	2	99.2%	< 0.0001	1.28 (0.79–2.07)	0.3137
Lag 3	1	NA		1.01 (1.00–1.01)	< 0.0001
Lag 4	1	NA		1.06 (1.05–1.08)	0.0033
**Model type**					
Multivariable analysis	3	99.3%	< 0.0001	0.99 (0.95–1.04)	0.6684
Univariable analysis	6	99.4%	< 0.0001	1.24 (1.11–1.39)	0.0001
**Overall**	12	99.3%	0	1.07 (1.04–1.11)	< 0.0001

**Table 6:** Subgroup analysis for the association between wind speed and scrub typhus

Subgroup types	n	Heterogeneity test		Pooled estimate	
		*I*^*2*^	*P*	RR (95%CI)	*P*
**Regions**					
China	6	98.6%	< 0.0001	0.60 (0.49–0.72)	< 0.0001
Laos	1	NA		0.52 (0.18–1.51)	0.228
**Lag group**					
Lag 0	1	NA		0.26 (0.22–0.30)	< 0.0001
Lag 1	3	0%	0.4059	0.97 (0.95–0.98)	< 0.0001
Lag 2	2	46%	0.1711	0.56 (0.44–0.70)	< 0.0001
Lag 3	1	NA		0.47 (0.31–0.71)	0.0003
**Model type**					
Multivariable analysis	4	0%	0.4005	0.58 (0.52–0.64)	< 0.0001
Univariable analysis	3	99.3%	< 0.0001	0.66 (0.53–0.81)	0.0001
**Overall**	7	99.3%	0	0.59 (0.49–0.71)	< 0.0001

**Table 7:** Subgroup analysis for the association between sunshine duration and scrub typhus

Subgroup types	n	Heterogeneity test		Pooled estimate	
		*I*^*2*^	*P*	RR (95%CI)	*P*
**Lag group**					
Lag 0	4	99.5%	< 0.0001	0.83 (0.66–1.05)	0.118
Lag 1	1	NA		0.87 (0.83–0.92)	< 0.0001
Lag 3	1	NA		1.39 (1.28–1.51)	0.0003
**Model type**					
Multivariable analysis	4	99.5%	< 0.0001	0.83 (0.66–1.05)	0.678
Univariable analysis	2	99.3%	< 0.0001	1.10 (0.69–1.74)	0.120
**Overall**	7	99.3%	0	0.92 (0.77–1.10)	0.345

##### Region group

Scrub typhus exhibits notable geographic specificity in its incidence, and in this meta-analysis, a large proportion of included studies were conducted in China. To further explore this aspect, we performed subgroup analyses by region, categorizing studies into two groups: those conducted in China and those from other countries (e.g., Korea, Laos, and India).

The subgroup analysis revealed that in the China-region group, the direction of meteorological effects was consistent with the overall results, with even stronger effect sizes observed. Notably, two studies from Korea demonstrated risk trends opposite to the overall effect in relation to land surface temperature (RR 0.97 vs RR 1.06). Although these findings did not reach statistical significance due to limited sample size, they suggest the possibility that rising land surface temperatures may suppress the ecological development of chigger mites in Korea.

Moreover, tests for between-group differences by country showed that the effect estimates for all meteorological factors differed significantly (*p* < 0.0001), indicating that geographic region may be a major source of heterogeneity.

##### Lag type

This study revealed notable temporal specificity in the response of scrub typhus incidence to meteorological exposures through stratified lag effect analyses.

Precipitation showed the strongest promotive effect in the same month of exposure (lag0: RR 1.05 vs overall: RR 1.01). In contrast, the maximum effects of temperature and relative humidity were observed at 1–2 months after exposure. Moreover, wind speed did not show significant effects across different lag periods.

The test for subgroup differences yielded statistically significant results (*p* < 0.0001), suggesting that meteorological exposures may exert biological lag-response effects, with the 1- to 2 month lag period being the most sensitive time window for scrub typhus incidence.

##### Model type

In this meta-analysis, we observed that univariable analysis tended to produce more pronounced or statistically significant relative risks compared to multivariable models, as seen in the cases of land surface temperature and relative humidity. In contrast, pooled estimates from multivariable analyses were generally attenuated and lacked statistical significance—for instance, with ambient temperature (*p* = 0.073), land surface temperature (*p* = 0.9309), precipitation (*p* = 0.3056), relative humidity (*p* = 0.6684), and sunshine duration (*p* = 0.678). Notably, in the subgroup analysis for relative humidity, the two modeling approaches yielded opposite effects: the univariable model suggested an increased risk (RR 1.24), whereas the multivariable model indicated a protective effect (RR 0.99).

Moreover, the test for between-group differences by model type revealed no statistically significant difference in effect sizes (*p* = 0.0981), suggesting that model type is unlikely to account for the observed heterogeneity. However, the discrepancy still highlights the potential for upward bias in univariable estimates due to unadjusted confounding. Multivariable analysis, which accounts for covariates, may provide more accurate approximations of the true effect—although limited statistical power may prevent these estimates from reaching significance.

#### Sensitivity analysis

To evaluate the robustness of the pooled effect estimates and identify potentially influential studies, we conducted a leave-one-out sensitivity analysis, whereby each study was excluded in turn and the meta-analysis was recalculated. The results showed that excluding any single study did not substantially alter the pooled relative risk (RR) or its 95%CI, indicating good robustness of the findings.

In summary, the sensitivity analysis demonstrated that the overall results were stable and not driven by any individual study.

#### Publication bias

In addition, we used funnel plots to assess the risk of publication bias for several meteorological factors (see Fig. 2). The funnel plots for ambient temperature, land surface temperature, and precipitation appeared relatively symmetric, suggesting a low likelihood of publication bias. In contrast, the funnel plot for relative humidity exhibited some asymmetry, indicating the potential presence of publication bias or small-study effects. Due to the limited number of studies available, funnel plots were not generated for wind speed, sunshine duration, and atmospheric pressure. However, further quantitative tests using Egger’s regression and Begg’s rank correlation did not detect significant evidence of systematic publication bias (*p* > 0.05). It should be noted that the statistical power of Egger’s and Begg’s tests is limited when fewer than ten studies are included.

In summary, the results of this meta-analysis are unlikely to be substantially influenced by publication bias.

### Dose–response meta-analysis results

To systematically evaluate the potential dose–response relationships of meteorological exposures, we integrated data from six studies [24, 31, 44–46] and conducted the dose–response meta-analysis on meteorological factors associated with scrub typhus. Due to the limited number of eligible studies, the analysis was restricted to three major meteorological variables: ambient temperature, precipitation, and relative humidity, summarizing the cumulative lagged relative risks across multiple exposure levels.

**Fig. 4:**
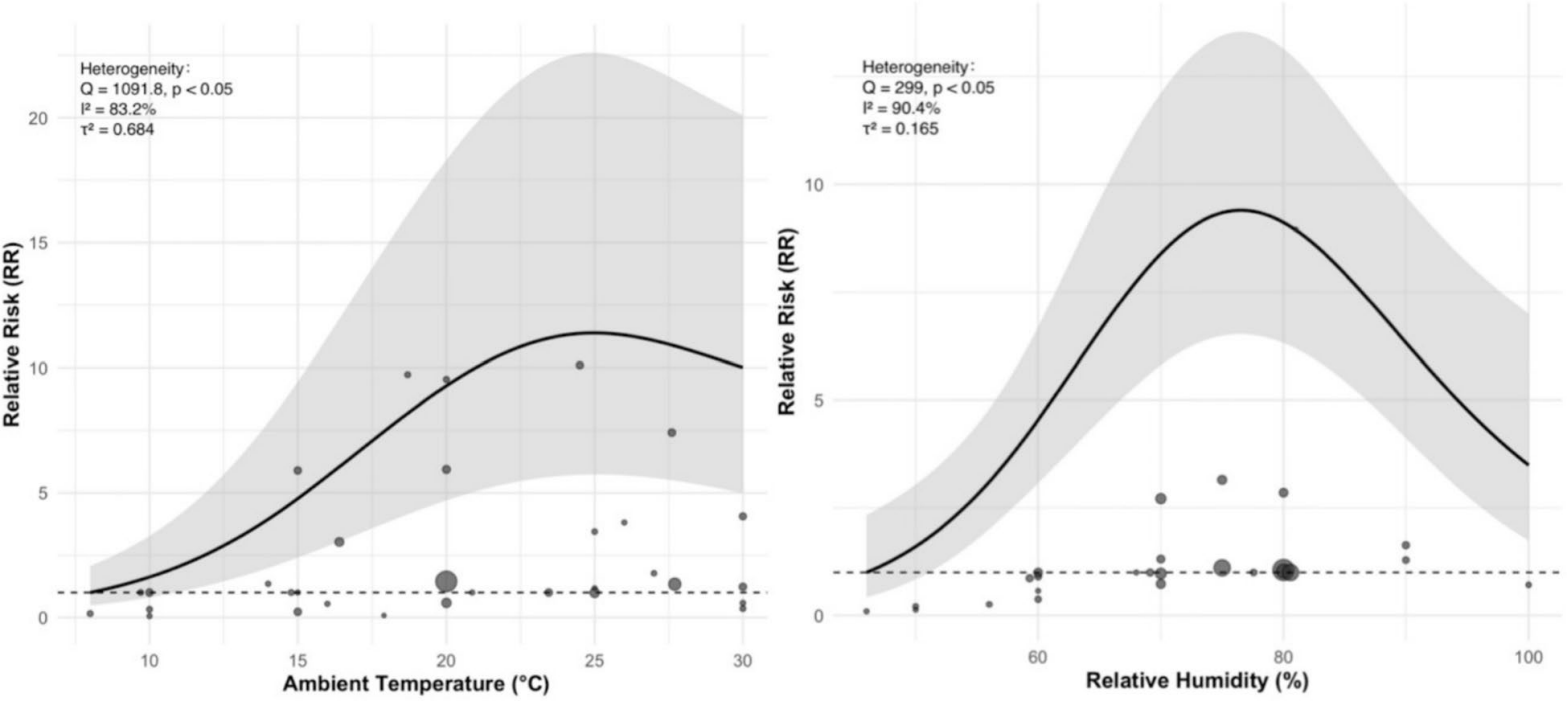
Nonlinear dose–response curves of ambient temperature and relative humidity with the risk of scrub typhus

The results demonstrated a significant nonlinear increasing trend in scrub typhus risk with rising ambient temperature, exhibiting an inverted U-shaped curve. The risk peaked at an average temperature of 25.02 °C (RR 11.391, 95% CI 5.740–22.604), followed by a notable decline (see Fig. 4 left).

Similarly, the association between relative humidity and scrub typhus incidence also showed an inverted U-shaped pattern. As humidity increased, the risk gradually rose, reaching its maximum at 76.4% relative humidity (RR 9.400, 95% CI 6.527–13.537), and then declined as humidity continued to rise (see Fig. 4 right).

**Fig. 5:**
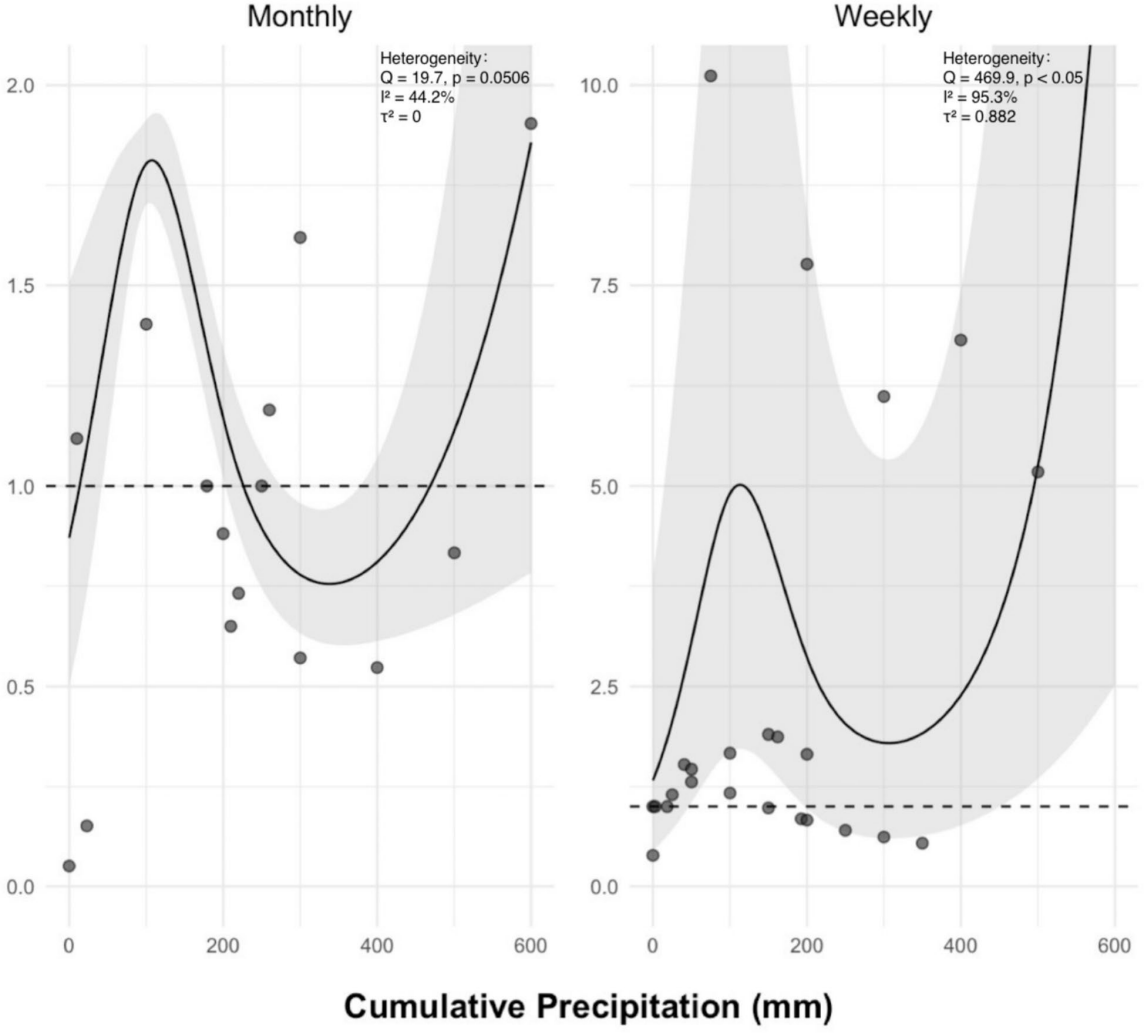
Nonlinear dose–response curves of cumulative precipitation with the risk of scrub typhus, stratified by exposure interval (monthly vs. weekly)

Considering the different time scales of data collection across studies, we stratified cumulative precipitation into weekly and monthly groups for separate analysis. Both groups exhibited a unimodal dose–response relationship (see Fig. 5), with a continuously increasing risk trend at higher precipitation levels. For monthly cumulative precipitation, the first peak in disease risk was observed at 109 mm (RR 1.81, 95% CI 1.70–1.93), followed by a gradual decline in risk, reaching a nadir at 338 mm (RR 0.756, 95% CI 0.606–0.943). In contrast, the curve for weekly cumulative precipitation was steeper, with a peak risk at 115 mm (RR 5.02, 95% CI 1.72–14.6), after which the risk declined but began to rise continuously beyond 308 mm (RR 1.79, 95% CI 0.603–5.33). Notably, when monthly precipitation exceeded 226 mm, further increases appeared to have a protective effect against scrub typhus, which contrasts with the consistently positive association observed for weekly precipitation. Moreover, weekly precipitation estimates showed wider confidence intervals. Although some inconsistencies were present, both analyses revealed a similar bimodal risk pattern.

This study observed substantial heterogeneity in the dose–response relationships for ambient temperature, relative humidity, and precipitation. Given the limited number of included studies (*n* = 6), most of which were conducted in China, with one conducted in Korea, subgroup analyses were not performed. To evaluate result robustness, we conducted sensitivity analyses. The results demonstrated that regardless of which individual study was excluded, the heterogeneity parameters *τ*^2^ (ambient temperature: 0.51–0.85; relative humidity: 0.18–0.24; precipitation: 0.19–0.91) and their derived *I*^*2*^ values (mean temperature: 78–90%; relative humidity: 37–63%; precipitation: 73–90%) fluctuated within reasonable ranges, indicating a balanced distribution of heterogeneity sources. Notably, the exclusion of Han 2023 [24] significantly reduced *τ*^*2*^ and I^2^ in the precipitation model, suggesting this study contributed substantially to precipitation-related heterogeneity. Crucially, however, the shape and trend of the nonlinear dose–response curve remained fundamentally unchanged.

In summary, the nonlinear characteristics (*p* < 0.001) of all three meteorological dose–response relationships persisted regardless of study exclusion, confirming that our dose–response meta-analysis results demonstrate high robustness and are not unduly influenced by individual studies.

In accordance with the established methodological consensus of the *Cochrane Handbook for Systematic Reviews of Interventions* [47] regarding the limited number of included studies (< 10), we abstained from statistical tests for publication bias or funnel plot construction to prevent potentially misleading conclusions due to insufficient statistical power.

## Discussion

As a climate-sensitive vector-borne disease, the transmission dynamics of scrub typhus are modulated by various meteorological factors. A previous systematic review [18] has explored the relationship between scrub typhus and environmental factors, including certain meteorological variables. However, the review primarily relied on qualitative synthesis and did not perform a meta-analysis.

In contrast, the present study, based on a systematic review and meta-analysis, quantitatively synthesized existing research data and systematically evaluated both the linear and nonlinear associations between various meteorological factors and the risk of scrub typhus incidence. This approach addresses the limitations of prior narrative reviews that lacked quantitative evidence and further enriches the empirical foundation for understanding the epidemiological link between meteorological factors and scrub typhus transmission.

In this systematic review and meta-analysis, we present updated evidence on the associations between multiple meteorological variables—including ambient temperature, land surface temperature, precipitation, relative humidity, wind speed, sunshine duration, and atmospheric pressure—and the risk of scrub typhus incidence. Subgroup analyses were also performed based on country, lag effects, and model type, yielding meaningful findings. In the following sections, we systematically elaborate on these results and attempt to interpret the potential mechanisms by which meteorological factors influence scrub typhus transmission, drawing upon geographical characteristics, differences in modeling approaches, and disease ecology.

### Temperature

In this study, we separately analyzed the effects of ambient temperature and land surface temperature on scrub typhus incidence. For each 1 °C increase in monthly average ambient temperature, the risk of scrub typhus increased by an average of 8%. This effect also exhibited a lag structure, with the highest risk observed at a one-month lag. The dose–response meta-analysis revealed an inverted U-shaped relationship between cumulative lagged ambient temperature and incidence. One study [24] described this as an “initial–elevated–descendent” pattern, with risk peaking at 25.02 °C before declining. This finding aligns with most existing epidemiological reports [24, 25].

Given the growing interest in land surface temperature, we also included it in our analysis. Each 1 °C increase in monthly average land surface temperature was associated with a 1% increase in risk, though the effect size was smaller compared to ambient temperature.

As the primary climatic driver of scrub typhus transmission, temperature regulates disease dynamics through dual pathways. Biologically, it influences chigger development, oviposition, and lifespan [48]. The optimal temperature range for chigger survival is 20–30 °C [26], within which their reproduction and survival activities are most vigorous. Moderate increases in temperature within this range can promote egg hatching and larval development; however, temperatures exceeding the thermal threshold can inhibit hatching rates, suggesting that extreme heat events may suppress scrub typhus transmission. Notably, *Leptotrombidium deliense*, the primary vector species in southern and eastern China, has been found to exhibit the highest field hatching rates at 23 ± 1 °C [49], closely aligning with the peak identified in our dose–response meta-analysis predominantly based on data from China. Moreover, ecological studies have shown that most chigger species display peak mobility at 22–24 °C [50], while rodent hosts, which serve as the primary reservoirs, exhibit heightened activity at 27–29 °C [51]. These findings further explain how temperature regulates the ecology of vectors and hosts, ultimately affecting disease transmission dynamics.

On the other hand, temperature significantly influences human behavior patterns in a nonlinear fashion. Rising temperatures lead to more outdoor activities, reduced clothing, and greater skin exposure, increasing contact with vectors. Additionally, previous studies have reported that extreme temperature events, such as severe cold or heatwaves, significantly reduce outdoor activity levels [52]. This nonlinear exposure pattern provides a behavioral explanation for the formation of the observed inverted U-shaped risk curve.

### Precipitation

Beyond temperature, precipitation is a critical meteorological driver in scrub typhus transmission. We found that each 1 mm increase in monthly cumulative precipitation significantly elevates the disease risk by 1%, with particularly pronounced immediate effects. Further dose–response meta-analysis demonstrated a nonlinear single-peak association between precipitation and cumulative lagged risk. The overall trend resembled a unimodal curve but showed a continuous increase at higher precipitation levels. Specifically, the disease risk initially rises with increasing precipitation, peaks at moderate levels, subsequently declines to a risk nadir at approximately 300 mm, but exhibits sustained elevation beyond this threshold, manifesting a distinct “high-precipitation tail effect”.

Notably, the precipitation amounts corresponding to peak risk were remarkably similar between weekly (115 mm) and monthly (109 mm) scales—a finding contradicting the theoretical expectation that monthly accumulations should quadruple weekly values. This paradox arises because the weekly peak reflects acute ecological disruptions from short-term torrential rainfall, whereas the monthly peak represents chronic cumulative effects of sustained moderate rainfall. Both mechanisms optimize transmission conditions but operate through distinct pathways. This mechanistic divergence is corroborated by risk curve characteristics: the steeper slope and wider confidence intervals of the weekly curve underscore the abrupt ecosystem disturbances induced by intense precipitation and thereby significantly impact disease risk.

Our findings align with prior research showing incidence peaks during rainy seasons. Moderate rainfall promotes vegetation growth and soil moisture, providing abundant food resources and optimal habitats for chiggers [53]. Additionally, it has been reported that the activity and transmission potential of *Leptotrombidium deliense* intensify during rainy periods, leading to higher attachment rates of chiggers on rodent hosts compared to other seasons. Conversely, excessive rainfall may damage habitats and lead to declines in both host and vector populations, thereby suppressing disease transmission [54]. Concurrently, increased rainfall limits human agricultural and outdoor activities, reducing exposure risk.

Some studies have also indicated that outbreaks of scrub typhus are associated with short-term extreme precipitation events [24]. Heavy rains and flooding can force rodents to migrate from natural habitats to human dwellings, while dislodging chiggers from vegetation, thereby increasing the likelihood of contact between chiggers, rodent hosts, and humans [23]. This mechanism aligns with our observation of a continuous increase in risk at the high end of the precipitation curve. Therefore, it is necessary to integrate public health interventions with meteorological early warning systems by establishing precipitation-based alerts, strengthening vector control during regular rainy seasons, and placing particular emphasis on personal protection during extreme rainfall events to reduce the risk of infection.

### Relative humidity

Our study demonstrates that each 1% increase in monthly mean relative humidity elevates scrub typhus risk by an average of 7%, with the most pronounced effect observed at a 2 month lag. Dose–response meta-analysis further revealed an inverted U-shaped nonlinear relationship between relative humidity and cumulative lagged risk: disease risk progressively increased with rising humidity, peaked at 76.4% relative humidity, and subsequently declined as humidity continued to rise. It is worth noting that a previous systematic review [18] also observed a potential W-shaped pattern in the association between relative humidity and scrub typhus incidence [55].

The observed pattern stems from humidity's fundamental regulation of chigger mite ecology. As chigger mites depend critically on environmental moisture for survival, humidity governs their geographical distribution [25] and influences rodent host reproductive dynamics [56]. Moderate humidity elevation creates the damp microhabitats preferred for mite reproduction and development. Empirical studies confirm optimal mite activity at > 50% humidity, whereas significant suppression occurs below 50% or above 75–80% [57].

Crucially, the 2-month lag effect aligns with the chigger mite life cycle. Humidity fluctuations during the preceding 1–2 months critically impact egg hatching success and larval survival. The life cycle of chiggers lasts approximately 2–3 months, which is longer than the incubation period of scrub typhus (averaging 10–12 days). Furthermore, the pathogen *Orientia tsutsugamushi* can be vertically transmitted to offspring through transovarial transmission in female chiggers [56]. It is therefore likely that humidity influences the density of the previous generation of chiggers [58], ultimately affecting disease transmission through the newly developed larval population two months later. This mechanism underscores how long-term meteorological factors indirectly influence scrub typhus transmission through multi-generational effects on vectors and hosts.

### Wind speed

This study identified strong wind conditions as a protective factor against scrub typhus incidence. For every 1 m/s increase in monthly average wind speed, the disease risk decreased by 41% on average. The underlying mechanisms remain incompletely understood, but it is hypothesized that strong winds may physically dislodge trombiculid mites from their favorable habitats, disrupting larval survival and development. This consequently reduces host attachment opportunities and disease transmission potential. Additionally, strong winds deter human outdoor activities, further diminishing exposure risk [59, 60].

However, these findings contradict certain existing studies reporting positive wind–typhus associations. A Chinese investigation [61] offers a potential explanation for this discrepancy: using a geographical detector model, it revealed a significant interaction effect between wind speed and forest coverage (*q* = 0.356). In areas with higher forest coverage, moderate to high wind speeds could facilitate the long-distance dispersal of chiggers on vegetation, thereby expanding the geographic range of disease transmission. Differences in vegetation cover across study areas may thus contribute to the observed inconsistencies.

Another Chinese study [62] demonstrated a nonlinear relationship: typhus risk initially increased with rising wind speeds but declined after exceeding 1.6 m/s.

These observations collectively indicate that wind influences scrub typhus transmission through complex, context-dependent pathways. Given the limited number of studies examining wind–typhus dose–response relationships, this research did not conduct corresponding meta-analyses.

### Sunshine duration

In this study, we identified sunshine duration as a potential protective factor for scrub typhus incidence, demonstrating that each 1 h increase in monthly mean sunshine duration was associated with an average 8% decreased risk of disease onset. However, this effect did not reach statistical significance. The limited statistical power may stem from insufficient included studies, potentially hindering detection of underlying effects. Notably, this finding contradicts prevailing research and a previous systematic review [18] suggesting sunshine duration acts as a risk factor. Unfortunately, no quantitative meta-analysis with adequate statistical power currently exists to resolve this controversy.

On one hand, longer sunshine duration may extend the time people spend engaging in outdoor activities, thereby increasing their exposure to chigger mites. On the other hand, it may also regulate vector biological rhythms. Evidence suggests that chigger mites exhibit a circadian activity pattern, with their activity peaking from the afternoon to sunset and declining to a low level at night, remaining low until sunrise. By concurrently extending human exposure windows and vector activity periods, sunshine duration collectively modulates scrub typhus transmission.

Although direct studies on sunshine duration's impact on mite ecology remain limited, relevant tick ecology research provides insight: evidence indicates [63] that photoperiod significantly affects tick development—long photoperiod conditions enhance reproductive capacity, whereas short photoperiod induces physiological suppression. Given biological similarities between ticks and trombiculid mites, future investigations should prioritize elucidating the underlying mechanisms in this context.

### Atmospheric pressure

In this study, we incorporated atmospheric pressure into our analysis. Each 1 hPa increase in monthly mean atmospheric pressure was associated with an average 6% elevation in scrub typhus risk. Although this association did not reach statistical significance, this trend aligns with most existing studies. The underlying mechanism may be attributed to the typical meteorological profile of high-pressure systems—characterized by lower humidity, reduced temperatures, and diminished sunshine duration—conditions collectively unfavorable for mite survival, thereby potentially suppressing scrub typhus transmission.

### Limitations

As the first meta-analysis to systematically quantify the relationship between scrub typhus and meteorological factors, this study provides a valuable evidence framework but also has inherent limitations. First, restricting inclusion to English-language publications may introduce the risk of language bias. Second, the number of eligible studies was limited and substantial heterogeneity was observed across studies; although subgroup and sensitivity analyses were performed to explore potential sources, their explanatory power remained insufficient. Due to data availability, incidence was adopted as the outcome measure. Variations in incidence definitions and measurement time scales across studies may have further contributed to heterogeneity, although the use of relative risks as the pooled effect size likely mitigated its impact. Moreover, results might differ if other outcomes, such as mortality or hospitalization, were considered. Future research should aim to establish standardized definitions of incidence and incorporate multiple outcome indicators to achieve a more comprehensive understanding. Third, most included studies were conducted in China, with limited data from other endemic regions, which may restrict the generalizability of the findings. Finally, the majority of studies were ecological in design, making residual confounding and ecological fallacy unavoidable. Therefore, the pooled effect estimates can only reflect associations at the population level and should not be directly extrapolated to individual risk.

## Conclusion

This study systematically evaluated both linear and nonlinear associations between multiple meteorological factors and scrub typhus incidence through linear and dose–response meta-analyses. We found that ambient temperature, land surface temperature, precipitation, relative humidity, and atmospheric pressure were positively associated with disease risk, whereas wind speed and sunshine duration showed negative associations. Nonlinear patterns, lag effects, and geographic differences were also identified, highlighting the complex pathways through which meteorological variability influences scrub typhus transmission. These findings provide quantitative evidence on the climatic drivers of scrub typhus, offering important implications for risk prediction, early warning, and disease prevention. Future research should expand to broader geographic regions and integrate multinational collaborative studies with climate modeling to further strengthen the scientific basis for predicting and controlling scrub typhus.

## Supplementary Information

Supplementary material 1. PRISMA 2020 Checklist.

Supplementary material 2.Table S1 Systematic literature review search terms and strategy; Table S2 Results of quality assessment using the Newcastle-Ottawa Quality Assessment System for studies included in the meta-analysis.

## Data Availability

The more detailed data used to support the findings of this study are available in the supplementary information.
