# Supplementary material for: Associations between meteorological factors and scrub typhus incidence: a systematic review and meta-analysis of linear and nonlinear dose–response relationships
Source: Trop Med Health. 2025 Oct 29;53:144. doi: 10.1186/s41182-025-00819-0 (PMC12570654; doi:10.1186/s41182-025-00819-0)
Supplement: Supplementary file 2 — Supplementary material 2.Table S1 Systematic literature review search terms and strategy; Table S2 Results of quality assessment using the Newcastle-Ottawa Quality Assessment System for studies included in the meta-analysis. [file 41182_2025_819_MOESM2_ESM.docx]

**Associations between meteorological factors and scrub typhus incidence: a systematic review and meta-analysis of linear and nonlinear dose–response relationships**

Shu Yang^1,2^ , Shu Yang^2^, Jun Guo^3^, Peng Li^4^, Yuling Xu^5^, Fei Hu^6^, Yiting Cui^1^, Ai Peng^1^, Yangqing Liu^2^, Yibing Fan^2^, Shihui Peng^2^, Hui Li^2,^ **^^[[1]](#footnote-1)^*^**, Peng Huang^1,^**^^[[2]](#footnote-2)^*^**

**Institutional and address:**

^^[[3]](#footnote-3)^^Center for Evidence-Based Medicine, Jiangxi Provincial Key Laboratory of Disease Prevention and Public Health, School of Public Health, Jiangxi Medical College, Nanchang University, Nanchang 330019, China

^^[[4]](#footnote-4)^^The Collaboration Unit for Field Epidemiology of State Key Laboratory of Infectious Disease Prevention and Control, Nanchang Center for Disease Control and Prevention, Nanchang 330038, China

^^[[5]](#footnote-5)^^Longnan Center for Disease Control and Prevention, Ganzhou 341700, Jiangxi, China

^^[[6]](#footnote-6)^^Nanfeng Center for Disease Control and Prevention, Fuzhou 344500, Jiangxi, China

^^[[7]](#footnote-7)^^Department of Cardiac Surgery, The Second Affiliated Hospital, Jiangxi Medical College, Nanchang Universcity, Nanchang 330000, China

^^[[8]](#footnote-8)^^Department of Cancer Medical Center, The First Affiliated Hospital, Jiangxi Medical College, Nanchang University, Nanchang 330019, China

**^[[9]](#footnote-9)^*Correspondence:** nccdcyjb@163.com; huangpengncu@163.com

**Table S1** Systematic literature review search terms and strategy

| **PubMed** |
| --- |
| ((((scrub typhus[MeSH Terms]) OR (Orientia tsutsugamushi)) OR (tsutsugamushi disease)) OR (tsutsugamushi Fever)) AND (((((((((((((((temperature[Title/Abstract]) OR (humidity[Title/Abstract])) OR (rain[Title/Abstract])) OR (rainfall,[Title/Abstract])) OR (precipitation[Title/Abstract])) OR (wind speed[Title/Abstract])) OR (wind velocity[Title/Abstract])) OR (sunshine[Title/Abstract])) OR (sunlight[Title/Abstract])) OR (atmospheric pressure[Title/Abstract])) OR (air pressure[Title/Abstract])) OR (barometric pressure[Title/Abstract])) OR (climate[Title/Abstract])) OR (weather[Title/Abstract])) OR (meteorolog*[Title/Abstract])) AND ("2015"[Date - Publication] : "2025"[Date - Publication]) |
| **Scopus** |
| ( ( TITLE-ABS-KEY ( temperature ) OR TITLE-ABS-KEY ( humidity ) OR TITLE-ABS-KEY ( rain ) OR TITLE-ABS-KEY ( rainfall ) OR TITLE-ABS-KEY ( precipitation ) OR TITLE-ABS-KEY ( wind AND speed ) OR TITLE-ABS-KEY ( wind AND velocity ) OR TITLE-ABS-KEY ( sunshine ) OR TITLE-ABS-KEY ( sunlight ) OR TITLE-ABS-KEY ( sunshine AND duration ) OR TITLE-ABS-KEY ( atmospheric AND pressure ) OR TITLE-ABS-KEY ( air AND pressure ) OR TITLE-ABS-KEY ( barometric AND pressure ) OR TITLE-ABS-KEY ( climate ) OR TITLE-ABS-KEY ( weather ) OR TITLE-ABS-KEY ( meteorolog* ) ) ) AND ( ( TITLE-ABS-KEY ( scrub AND typhus ) OR TITLE-ABS-KEY ( orientia AND tsutsugamushi ) OR TITLE-ABS-KEY ( tsutsugamushi AND disease ) OR TITLE-ABS-KEY ( tsutsugamushi AND fever ) ) ) AND PUBYEAR > 2014 AND PUBYEAR < 2026 |
| **Web of Science** |
| **#1:** (((TS=(Scrub Typhus)) OR TS=(Orientia tsutsugamushi)) OR TS=(Tsutsugamushi Disease)) OR TS=(Tsutsugamushi Fever) and Preprint Citation Index (Exclude – Database) |
| **#2:** ((((((((((((((TS=(temperature)) OR TS=(humidity,)) OR TS=(rain)) OR TS=(rainfall)) OR TS=(precipitation)) OR TS=(wind speed)) OR TS=(wind velocity)) OR TS=(sunshine)) OR TS=(sunlight)) OR TS=(atmospheric pressure)) OR TS=(air pressure)) OR TS=(barometric pressure)) OR TS=(climate)) OR TS=(weather)) OR TS=(meteorolog*) and Preprint Citation Index (Exclude – Database) |
| **#1** AND **#2** and Preprint Citation Index (Exclude – Database) and 2015 or 2016 or 2017 or 2018 or 2019 or 2020 or 2021 or 2022 or 2023 or 2024 or 2025 (Publication Years) |
| **EMBASE** |
| **#1:** 'scrub typhus':ti,ab,kw OR 'orientia tsutsugamushi':ti,ab,kw OR 'tsutsugamushi disease':ti,ab,kw OR 'tsutsugamushi fever':ti,ab,kw |
| **#2:** temperature:ti,ab,kw OR humidity:ti,ab,kw OR rain:ti,ab,kw OR rainfall:ti,ab,kw OR precipitation:ti,ab,kw OR 'wind speed':ti,ab,kw OR 'wind velocity':ti,ab,kw OR sunshine:ti,ab,kw OR sunlight:ti,ab,kw OR 'sunshine duration':ti,ab,kw OR 'atmospheric pressure':ti,ab,kw OR 'air pressure':ti,ab,kw OR 'barometric pressure':ti,ab,kw OR climate:ti,ab,kw OR weather:ti,ab,kw OR meteorolog*:ti,ab,kw |
| **#1** AND **#2** |

**Table2 Results of quality assessment using the Newcastle-Ottawa Quality Assessment System for studies included in the meta-analysis**

| Study | Selection | | | | Comparability | Outcome | | | Score | quality |
| --- | --- | --- | --- | --- | --- | --- | --- | --- | --- | --- |
|  | Representativeness of the exposed cohort | Selection of the non exposed cohort | Ascertainment of exposure | Demonstration that outcome of interest was not present at start of study | Comparability of cohorts on the basis of the design or analysis | Assessment of outcome | Was follow-up long enough for outcomes to occur | Adequacy of follow up of cohorts |  |  |
| Wu 2016 | **★** | ☆ | **★** | ☆ | **★**☆ | **★** | **★** | **★** | 6/9 | moderate |
| Sun 2017 | **★** | **★** | **★** | **★** | **★**☆ | **★** | **★** | ☆ | 7/9 | high |
| Wei 2017 | ☆ | **★** | **★** | **★** | **★**☆ | **★** | **★** | ☆ | 6/9 | moderate |
| Kang 2018 | **★** | **★** | **★** | **★** | **★**☆ | **★** | **★** | ☆ | 7/9 | high |
| Kim 2018 | **★** | ☆ | **★** | **★** | **★**☆ | **★** | **★** | **★** | 7/9 | high |
| Yao 2019 | **★** | ☆ | **★** | **★** | **★★** | **★** | **★** | ☆ | 7/9 | high |
| Lu 2021 | ☆ | ☆ | **★** | **★** | **★**☆ | **★** | **★** | **★** | 6/9 | moderate |
| Roberts 2021 | **★** | ☆ | **★** | **★** | **★**☆ | **★** | **★** | **★** | 7/9 | high |
| Luo 2022 | **★** | **★** | **★** | **★** | **★**☆ | **★** | **★** | **★** | 8/9 | high |
| Han 2023 | **★** | **★** | **★** | **★** | **★**☆ | **★** | **★** | **★** | 8/9 | high |
| Li 2023 | ☆ | **★** | **★** | **★** | **★★** | **★** | **★** | ☆ | 7/9 | high |
| Wei 2023 | ☆ | **★** | **★** | **★** | **★★** | **★** | **★** | **★** | 8/9 | high |
| Chang 2024 | **★** | ☆ | **★** | **★** | **★★** | **★** | **★** | ☆ | 7/9 | high |
| D'Cruz 2024 | ☆ | ☆ | **★** | **★** | **★★** | **★** | **★** | ☆ | 6/9 | moderate |
| Luo 2024 | ☆ | ☆ | **★** | **★** | **★**☆ | **★** | **★** | ☆ | 5/9 | moderate |
| Pan 2024 | **★** | ☆ | **★** | **★** | **★**☆ | **★** | **★** | **★** | 7/9 | high |
| Qian 2024 | **★** | **★** | **★** | **★** | **★★** | **★** | **★** | **★** | 9/9 | high |

1. [↑](#footnote-ref-1)
2. [↑](#footnote-ref-2)
3. [↑](#footnote-ref-3)
4. [↑](#footnote-ref-4)
5. [↑](#footnote-ref-5)
6. [↑](#footnote-ref-6)
7. [↑](#footnote-ref-7)
8. [↑](#footnote-ref-8)
9. [↑](#footnote-ref-9)
